# The use of case studies to drive bottom-up leadership in community-oriented integrated care and health promotion (COIC)

**DOI:** 10.1080/17571472.2016.1271497

**Published:** 2017-01-09

**Authors:** John Sanfey

**Affiliations:** NHS England, North-West London

**Keywords:** Community-oriented integrated care, case studies, reflective practice, reflexivity, wicked problems, primary care, social care, health promotion, community

## Abstract

*London Journal of Primary Care* is supporting a collaborative network of multidisciplinary colleagues with an interest in community-oriented health care and health promotion (COIC). Case study methodology is well suited to generating knowledge from the frontline of health and social care service delivery and is a much under-developed resource. It is most effective when dealing with *wicked* problems, namely, the sort of complex, entangled and multi-faceted problems that successful COIC programmes must overcome. Used collaboratively, it supports effective networking across professional and community boundaries.

## Why this matters to me

Case study methodology adds an additional dimension to reflective practice, which empowers professionalism and bottom-up leadership.

## Key message

Case study methodology is well suited to the task of enabling multidisciplinary teams on the ground, together with their communities, to develop and share knowledge relating to the integration of social, health and preventive health on the ground.

‘The search for scientific bases for confronting problems of social policy is bound to fail, because of the nature of these problems. They are ‘wicked’ problems, whereas science has developed to deal with ‘tame’ problems’ [1].

The recent Five-Year, and GP Forward Views call for better inter-disciplinary collaboration and integration across the boundaries of health and social care. Primary health care is to become more proactive: to forge links with community-based projects and charities and develop what this journal calls community-oriented integrated care and health promotion (COIC) [2,3]. Not least among the challenges is the overstretched condition of the primary and social care workforces who are saturated with top-down initiatives. Perhaps a more fundamental problem is that the scientific paradigm familiar to health professionals is not well suited to the domain of social policy and planning.

Problems related to social planning are frequently cited as examples of *wicked* problems [1]. Other examples include terrorism and environmental degradation [4]. Each one is a complex set of entangled conundrums. Wicked problems are not strictly solvable and have no single right or wrong answer. The goal is to improve matters by developing models of understanding that permit some generalisation and inspire shared learning. Wicked problems can only be formulated in terms of a particular solution being considered, unlike traditional science where problems can in principle be formulated into answerable questions. For example, the integration of care in COIC is a solution, but it is difficult to describe many aspects of the problem being solved except in terms of fragmented services and communities. Investigators examining a wicked problem must make choices that determine the shape of the enquiry and any solutions proposed. This is called *reflexivity* [5]. Each intervention re-shapes the original problem and its context, which makes the exact replication of interventions impossible.

Case study methodology is well suited to the exploration of wicked problems, and various authors have called for it to be used more widely in primary care [6,7]. Wicked problems generally require creative, innovative solutions, and case study methodology comes in many varieties. Indeed, one of its great strengths is the flexibility to design a bespoke study to explore a particular issue [8]. Furthermore, problems are explored in their own settings, unlike in traditional research where the experimental design will usually include an attempt to control the setting, as for example in a randomised controlled trial [7]. Case study methodology works best when teasing out complex issues, identifying patterns and developing models in what Schon calls the *swampy lowlands* in which professionals operate [9]. Perhaps even more important in the context of a demoralised workforce, is that case studies can readily be conducted by those delivering services, in partnership with the citizens who benefit from them. Participation in the research process is especially empowering for teams and their communities; it also provides tools to re-shape their services with locally-generated evidence. Chris Ham and others have long argued for bottom-up leadership in health care [10]. Sanfey and Ahluwalia suggested the principle of *earned autonomy* be applied to individuals, teams and systems that can effectively self-correct [11]. Case study methodology provides the means to achieve this, building on professional reflective practice to develop techniques, changes to practice and new models based on rapid-cycle testing and the formal peer-review of case study evidence.

The data generated by case studies can be very diverse, as well as the problems and settings being studied, and the professionals and communities involved. In an excellent review article, Sarah Crowe and colleagues provide a good overview of case study methodology with its various methods of data analysis [7]. The simplest form will be familiar to most health professionals. GPs in particular are expected to include at least two cases in their annual appraisal. At present, most of the cases in an appraisal are basic descriptions with a few simple observations made by the doctor as researcher. Many of them however, also include some quality improvement, sometimes system-wide, with actions that arise from reflection on particular cases. The very best contain cycles of reflection-innovation-audit [12]. The potential of case study research is even greater than this, even for single cases with a lone researcher. Much of the potential lies in the further investigation of issues arising from reflection on single cases.

One simple way to categorise case study design is based on whether a case itself is unique, or whether it is chosen as the instrument to examine a particular phenomenon. The former has been called *intrinsic* case study, and the latter *instrumental* [8]. Often, a unique case will raise questions that warrant further investigation, and an intrinsic case study becomes an instrumental one. Consider the following example:

Suppose during a multidisciplinary team (MDT) meeting a practice nurse describes meeting someone regarded as a vulnerable adult, who reported a positive experience of an informal film club in their hostel. The man in question subsequently became much more confident and was able to live and thrive independently. Some of the other hostel residents also loved the film club but others drifted away over time. She describes the case to her colleagues. So far, this is a single, *intrinsic* case study. The MDT group thinks about developing a project to invite vulnerable young adults with mental health problems to a weekly film club discussion. At this point they have developed a model, namely, a film club for vulnerable adults. Now, the issue is no longer about the person as a unique event but rather about the need to explore the potential of their idea. They set about designing an instrumental case study, in which the subject of study is their model, namely, the phenomenon of a film club experience on vulnerable adults. They consider only speaking to the various people who found the film club to be positive including the original man who saw the practice nurse. However, they realise that since they have a model in mind and are pretty keen to implement it, the task is to challenge the model and perfect it. The commonest mistake people make when developing and testing innovative models is to seek evidence to confirm the value of the model they are testing [13]. The concept of *confirmation bias* arises from Karl Poppers seminal work on the *falsification thesis* as the basis of scientific method [14]. With this in mind, the group decides to speak to all the people involved, *especially* those who had drifted away from that particular film club. In other words, rather than seeking data to confirm the value of the model they are proposing, they deliberately seek out evidence that threatens their model. Armed with this evidence they are much more likely to improve their model before it is implemented. A truly useful model will be the better for surviving the challenge, and a useless one quickly put to rest.

The value of this kind of case study depends on having a sensible, clear description of the reflexive journey taken by the researchers. The process is clearly qualitative. Different MDT teams might have made different decisions. The value of the published research requires a judgement by the reader on the decisions taken by the investigator.

*Collective* case studies represent perhaps the most exciting route to system-wide development based on collaborative reflective practice in the field of COIC. They can take many forms, ranging a collection of simple intrinsic cases, to the comparison of complex instrumental studies across multiple sites. They can be simple, descriptive comparisons seeking to find patterns, hypotheses etc. from which to make generalizable inferences and recommendations. Alternatively, they can become a prolonged iteration of cyclical interventions and re-assessments in order to develop complex multi-system changes. The Health Foundation examination of seven networks an example of the former [15]. This case study explored the qualities of successful networks. Data were derived from published literature about the networks and from interviews with people involved. They developed the *Five*-*Cs wheel* model that measures the effectiveness of any network, and they felt confident enough in their methodology to recommend its widespread application. Incidentally, the five Cs in question are: co-operative structure, collective intelligence, critical mass, collective intelligence and, most essentially, common purpose.

Thomas and his colleagues went further than The Health Foundation in an example of a collective, comparative case study with interventions along the way and frequent re-evaluation. The study explored ways of increasing the capacity for innovation in primary care organisations [16]. They chose to form a sustained association between four teams over a period of three years. They embarked on a cyclical, iterative process that included three annual conferences to share experiences with workshops to develop particular ideas. There were also several changes of directions along the way before they felt able to draw conclusions from their journey.

In the interest of developing the field of COIC, *London Journal of Primary Care* will seek to publish from across the whole spectrum of case study methodology of single, intrinsic cases to multi-site comparative case studies. We also invite secondary research papers that simply compare and contrast published work relating to COIC. We are keen to explore how various project teams and organisations set about this important area, especially new care models such as multispecialty community providers integrating out-of-hospital care based on general practice and Accountable Care Organisations aligning general practice and hospital services. The Landscape section of the Journal will publish short, less formal, reflective pieces that might simply be commentaries or critiques of published work.

In summary, case study research is uniquely suited to developing models of community oriented integrated care and health promotion. The Health Foundation, citing earlier work by Ferlie et al. [17], described how multi-disciplinary networks are particularly well suited to tackling wicked problems [15]. Collaborative case study methodology can empower front-line staff across the boundaries of the various community-based disciplines by enabling them to innovate and drive policy based upon collaborative reflective practice. This Journal is launching a sustained initiative to promote case study research and support the development of collaborative, community-based networks. We will seek to provide both a forum and the necessary resources to achieve this. We invite individuals, teams, organisations and communities to contact us for support in developing case studies and to share knowledge and resources.

## Disclosure statement

No potential conflict of interest was reported by the author.
